# Additional value and new insights by four-dimensional flow magnetic resonance imaging in congenital heart disease: application in neonates and young children

**DOI:** 10.1007/s00247-020-04885-w

**Published:** 2020-12-11

**Authors:** Julia Geiger, Fraser M. Callaghan, Barbara E. U. Burkhardt, Emanuela R. Valsangiacomo Buechel, Christian J. Kellenberger

**Affiliations:** 1 Department of Diagnostic Imaging, University Children’s Hospital Zürich, Steinwiesstr 75, 8032 Zürich, Switzerland; 2 Children’s Research Centre, University Children’s Hospital Zürich, Zürich, Switzerland; 3 Center for MR research, University Children’s Hospital Zürich, Zürich, Switzerland; 4 Department of Pediatric Cardiology, University Hospital Zürich, Zürich, Switzerland

**Keywords:** Children, Congenital heart disease, Four-dimensional flow, Heart, Magnetic resonance imaging, Neonates

## Abstract

Cardiovascular MRI has become an essential imaging modality in children with congenital heart disease (CHD) in the last 15–20 years. With use of appropriate sequences, it provides important information on cardiovascular anatomy, blood flow and function for initial diagnosis and post-surgical or -interventional monitoring in children. Although considered as more sophisticated and challenging than CT, in particular in neonates and infants, MRI is able to provide information on intra- and extracardiac haemodynamics, in contrast to CT. In recent years, four-dimensional (4-D) flow MRI has emerged as an additional MR technique for retrospective assessment and visualisation of blood flow within the heart and any vessel of interest within the acquired three-dimensional (3-D) volume. Its application in young children requires special adaptations for the smaller vessel size and faster heart rate compared to adolescents or adults. In this article, we provide an overview of 4-D flow MRI in various types of complex CHD in neonates and infants to demonstrate its potential indications and beneficial application for optimised individual cardiovascular assessment. We focus on its application in clinical routine cardiovascular workup and, in addition, show some examples with pathologies other than CHD to highlight that 4-D flow MRI yields new insights in disease understanding and therapy planning. We shortly review the essentials of 4-D flow data acquisition, pre- and post-processing techniques in neonates, infants and young children. Finally, we conclude with some details on accuracy, limitations and pitfalls of the technique.

**Electronic supplementary material:**

The online version of this article (10.1007/s00247-020-04885-w) contains supplementary material, which is available to authorized users.

## Introduction

Cardiovascular MRI has become a complementary modality to echocardiography and a valuable alternative to conventional X-ray angiography and CT in neonates and young children with congenital heart disease (CHD) for preoperative assessment of cardiovascular anatomy and function and for follow-up after interventions or cardiovascular surgery [1–4]. Even if echocardiography still represents the first-line modality for diagnosing CHD, its quality can be limited by individual acoustic window [5]. Both CT and catheter angiography are burdened by ionising radiation that should be kept to a minimum in children. Thus, if complete diagnosis cannot be achieved by echocardiography, MRI is the ideal second-line technique because it provides combined information of anatomy and haemodynamics.

Four-dimensional (4-D) flow MRI was added to the range of available MR sequences almost a decade ago. It has been used for improved cardiovascular assessment, particularly for the heart and the aorta. In recent years, there has been an increasing number of 4-D flow MRI publications about young patients with complex CHD [6–15] and aortic pathologies [16–21]; however, the reported cohorts consist mainly of very heterogeneous collectives with regard to types of cardiovascular pathology as well as patient demographics. Among these publications, some aimed to assess the course of the disease by performing follow-up scans [18, 20]. Because 4-D flow MRI enables flow visualisation and quantification within a large three-dimensional (3-D) volume covering almost the entire thorax, its application in paediatric CHD helps to increase our understanding of the mechanisms and the significance of abnormal blood flow patterns. It might also significantly contribute to surgical and interventional planning and provide some help for risk stratification. Few publications have investigated the application of 4-D flow MRI in young children, many of them using ferumoxytol as a contrast medium [6, 22–24].

In this article, we illustrate indications for 4-D flow MRI applications and its clinical value in neonates, infants and young children with complex CHD and aortic pathologies preoperatively as well as in post-surgical conditions. Furthermore, we demonstrate some examples in which 4-D flow MRI provided valuable information concerning shunt quantification in cases with abnormal vasculature. We would like to show readers that even in neonates, 4-D flow MRI can be performed with excellent image quality if the sequence parameters are adjusted to the small cardiovascular dimensions. In addition, we briefly discuss important technical considerations regarding data acquisition, measurement correction and post-processing options, including the technique’s accuracy, and some methodological limitations and pitfalls.

## Conventional cardiovascular magnetic resonance examination

We usually examine neonates and young children under general anaesthesia with intubation in order to obtain cine images and contrast-enhanced angiography in breath-hold; these images show better quality compared with images acquired during spontaneous breathing under sedation. For safety reasons, i.e. secured airways in at-risk patients, our departmental anaesthesiology rule requires general anaesthesia for MR imaging in small children with complex CHD. Nonetheless, cardiovascular imaging protocols should be as short as possible to minimise potential complications related to anaesthesia. The following descriptions are based on our experience using a 1.5-tesla (T) scanner (Discovery MR 450; GE Healthcare, Waukesha, WI) and a multichannel surface coil fitting the child’s chest. The field of view should be adapted to the baby’s size. Matrix parameters and slice thickness should be adjusted for all sequences to achieve a high spatial resolution for depiction of the small cardiovascular structures.

Our MRI protocol for neonates is rather short and mainly consists of cine steady-state free precession sequences in multiple planes and contrast-enhanced magnetic resonance (MR) angiography or anatomical imaging as described by Kellenberger et al. [1]. Electrocardiogram (ECG)-gated two-dimensional (2-D) cine steady-state free precession sequences in the 2- and 4-chamber views as well as in the short axis are crucial for assessing cardiac morphology and function. We add axial slices covering the chest in many cases, particularly in the child’s first scan with an unclear anatomy. High-spatial-resolution imaging is mandatory for visualising the abnormal cardiovascular anatomy in small children. At our institution, we perform MR angiography with submillimetre resolution for this purpose using a double dose of a macrocyclic gadolinium-based contrast medium and automated bolus detection. Other institutions have used ferumoxytol enhancement for improved vascular imaging of neonates and infants with CHD because of longer residence in the blood pool [23–25]. However, ferumoxytol is not approved for application as a contrast agent and is not available everywhere for off-label use.

Blood flow quantification is an integral part of the diagnostic workup for children with CHD because it adds haemodynamic information to the anatomical images. Flow measurements enable quantification of shunts (pulmonary-to-systemic blood flow ratio) and of differential lung perfusion (blood flow to the right versus the left pulmonary artery) [26, 27]. The conventional 2-D velocity-encoded phase-contrast measurements require direct on-site supervision by an experienced cardiac imager to obtain adequate imaging data and accurate flow measurements. Correct plane placement can be challenging, in particular in neonates and small children because of their small vessel size and in the presence of complex vascular anatomy. Furthermore, acquisition of multiple 2-D phase-contrast measurements can be time-consuming in individual cases.

A conventional imaging protocol for CHD usually includes 2-D phase-contrast cine images in the ascending aorta, main pulmonary artery, and right and left pulmonary arteries. In particular cases, such as after Fontan palliation, additional flow measurements in the systemic or pulmonary veins are needed. Table 1 gives an overview of the most important parameters of the sequences we use for cardiovascular imaging in neonates and young children.

**Table 1:** Selected technical parameters for the most important sequences in cardiovascular MRI in neonates and young children

Parameter	4-D flow MRI	2-D phase contrast	Cine SSFP	MR angiography
Breath-hold	No	If possible	Yes	Yes
Gating	ECG	ECG	ECG	No
Repetition time (ms)	4.3–4.5	6.6	4.0	3.7
Echo time (ms)	2.5	3.6	1.7	1.5
Field of view (mm)	250–320	180–240	200–240	220–300
Matrix	160–200×160–200	256×128	192×224	320×160–224
Slice thickness (mm)	1.6–1.8	4.0	4.0–5.0	1.6–2.4
Views per segment	2	6	8/16	–
Flip angle (°)	15	20	45	30

*4-D* four-dimensional, *ECG* electrocardiogram, *MR* magnetic resonance, *MRI* magnetic resonance imaging, *SSFP* steady-state free precession

## Four-dimensional flow magnetic resonance imaging — acquisition

Currently used 4-D flow MRI sequences differ between Cartesian and non-Cartesian (i.e. spiral or radial) acquisitions [28–34]. Ongoing developments in recent years have considerably reduced scan times by parallel imaging techniques and compressed sensing. Recent publications report scan times of 2 min using k-space and time acceleration [35–39]. Schrauben et al. [34] achieved short scan times for neonates on the order of 3 min by using a motion-robust and respiratory-resolved 3-D radial flow acquisition that addresses the need for fast, high-resolution imaging in neonates with CHD.

Four-dimensional flow MRI is easier to plan than 2-D phase contrast because 4-D flow MRI acquires a full 3-D volume and does not require detailed knowledge of the course of each individual vessel during scanning. The 3-D volume should cover the entire heart and all the vessels of interest. Shim coverage, slice thickness, velocity encoding and spatial and temporal resolution can vary depending on the sequence and the scanner type, and should be adapted to the child’s size and heart rate.

Generally agreed-upon recommendations for acquisition of 4-D flow MRI have been published in a consensus statement written by international experts [40]. More specific recommendations for intracardiac application in CHD are reported by the International Society for Magnetic Resonance in Medicine flow and motion study group in a more recent publication [41]. In addition to these guidelines, one should keep in mind that the physiological specifics of small children require appropriate adaptions.

The 4-D flow sequence we use in our centre is a short echo time (TE) and repetition time (TR) radiofrequency-spoiled gradient-echo sequence accelerated by kt-ARC, a spatiotemporal-correlation-based auto-calibrating parallel imaging method. This currently results in acquisition times of 5–8 min depending on the heart rate. We usually acquire the 4-D flow MRI sequence at the end of the examination after contrast application for MR angiography so that we can benefit from the contrast-based increase in signal-to-noise ratio in magnitude data and noise reduction in velocity data. The sequence is acquired with retrospective ECG-gating and in free-breathing with inherent respiratory compensation.

We usually perform the 4-D flow acquisition in axial orientation covering the complete heart and the aortic arch. For assessing the small hearts and vessels of neonates, we acquire at an isotropic spatial resolution of 1.6 mm^3^ that can be increased to 1.8 mm^3^ in infants and young children (corresponding reconstructed in-plane resolution 1.2 mm^2^ and 1.4 mm^2^). The low signal-to-noise ratio from such small voxel sizes is compensated by using a multi-channel surface coil. We target a temporal resolution between 20 ms and 25 ms; this is achieved by adapting the number of phases to the individual heart rate. With fast heart rates in neonates up to 150 beats per minute we aim at a temporal resolution below 20 ms and 20–25 ms with lower heart rates in young children (~100 beats per minute). The default velocity encoding setting is 160 cm/s. Velocity encoding should be increased to 200 cm/s or more if higher velocities are expected on the basis of the anatomical findings or the available echocardiographic report. Detailed sequence parameters are shown in Table 1.

## Four-dimensional flow magnetic resonance imaging — post-processing

Several home-built as well as commercial software packages are available for editing and post-processing 4-D flow data. Among the commercially available sequences are Circle cvi42 (Circle Inc., Calgary, Canada), CAAS MR Solutions (Pie Medical Imaging, Maastricht, The Netherlands), Arterys Cardio AI^MR^ (Arterys Inc., San Francisco, CA) and MEDIS QFlow 4D (Medis Medical Imaging Systems, Leiden, The Netherlands). Different flow visualisation options are offered: the most commonly used are velocity magnitude maps, colour-coded streamlines, and velocity vectors complemented by pathline tracking that is usually performed based on vessel origin [28, 40].

For flow quantification, background phase correction is necessary [42]; standard automated/semi-automated procedures facilitate the static tissue identification; however, in small children accuracy of delineation should be verified. Locations with turbulent or very fast flow can be identified on the velocity colour map images; measurements in these areas should be avoided because of the potential for signal drop to result in incorrect quantification. Some post-processing software also offers the ability to unwrap small areas of phase aliasing. If this tool is not available and peak velocities cannot be changed, flow should be measured proximally or distally to the area of aliasing.

At our institution and for clinical routine, we use cloud-based post-processing software (Arterys) for flow assessment and visualisation. It allows for background phase correction, interactive panning and rotation, multiplane reconstructions of the three-dimensional volume and temporal visualisation of velocity magnitude maps, streamlines and velocity vector fields. Four-dimensional flow enables retrospective definition of all required measurements for comprehensive flow assessment; the ability to place all desired planes during post-processing is one of the major advantages of 4-D flow compared to 2-D phase contrast. Correct plane positioning perpendicular to the flow direction or vessel course is achieved by using three-plane images and double oblique technique similar to 2-D phase contrast. Planes can be placed manually and have to be adapted manually or semi-automatically to the vessel boundaries during the heart cycle. Valve tracking, particularly of the atrioventricular valves, should be performed for precise flow quantification at the level of the valves because they can move considerably between systole and diastole [43–45].

During a typical clinical workflow, flow volumes in the ascending aorta and the main, right and left pulmonary arteries are assessed within less than 10 min of post-processing time. Additional flow measurements can be added anytime, whenever needed. The sum of all individual pulmonary veins can be used as additional assessment for pulmonary blood flow volume, for instance in the presence of stents in the pulmonary arteries or for internal validation. In Fontan patients, flow from the systemic veins is typically measured and should correspond to the pulmonary blood flow volume. The results of quantitative flow analysis include information on forward and backward flow as well as peak velocity and allow calculation of regurgitation fraction and estimation of pressure gradients over stenoses.

In addition to the cloud-based application, we use in-house-developed routines for pathline tracking and assessment. Pathline analysis tracks the movement of blood flow over a cardiac cycle and permits the visualisation of shunts as well as the quantification of flow distribution.

## Four-dimensional flow magnetic resonance imaging — indications and benefits

Four-dimensional flow MRI can be beneficial in many congenital cardiovascular lesions. In the following sections, we present different indications and clinical examples in which 4-D flow MRI has added valuable haemodynamic information to the standard cardiac MRI examination.

### Flow visualisation

Flow visualisation in all three dimensions enables a quick overview of flow velocity distribution and direction in the entire heart and any thoracic vessel except for the coronaries. Flow visualisation simplifies correct perpendicular plane positioning and improves quantitative flow assessment because areas with flow turbulence can be avoided. Moreover, by using different visualisation options, detection of flow abnormalities becomes very intuitive, unlike with standard cardiac MRI sequences. Direct visualisation of flow velocity and directional changes over the cardiac cycle is an outstanding advantage compared to conventional MRI techniques.

**Fig. 1:**
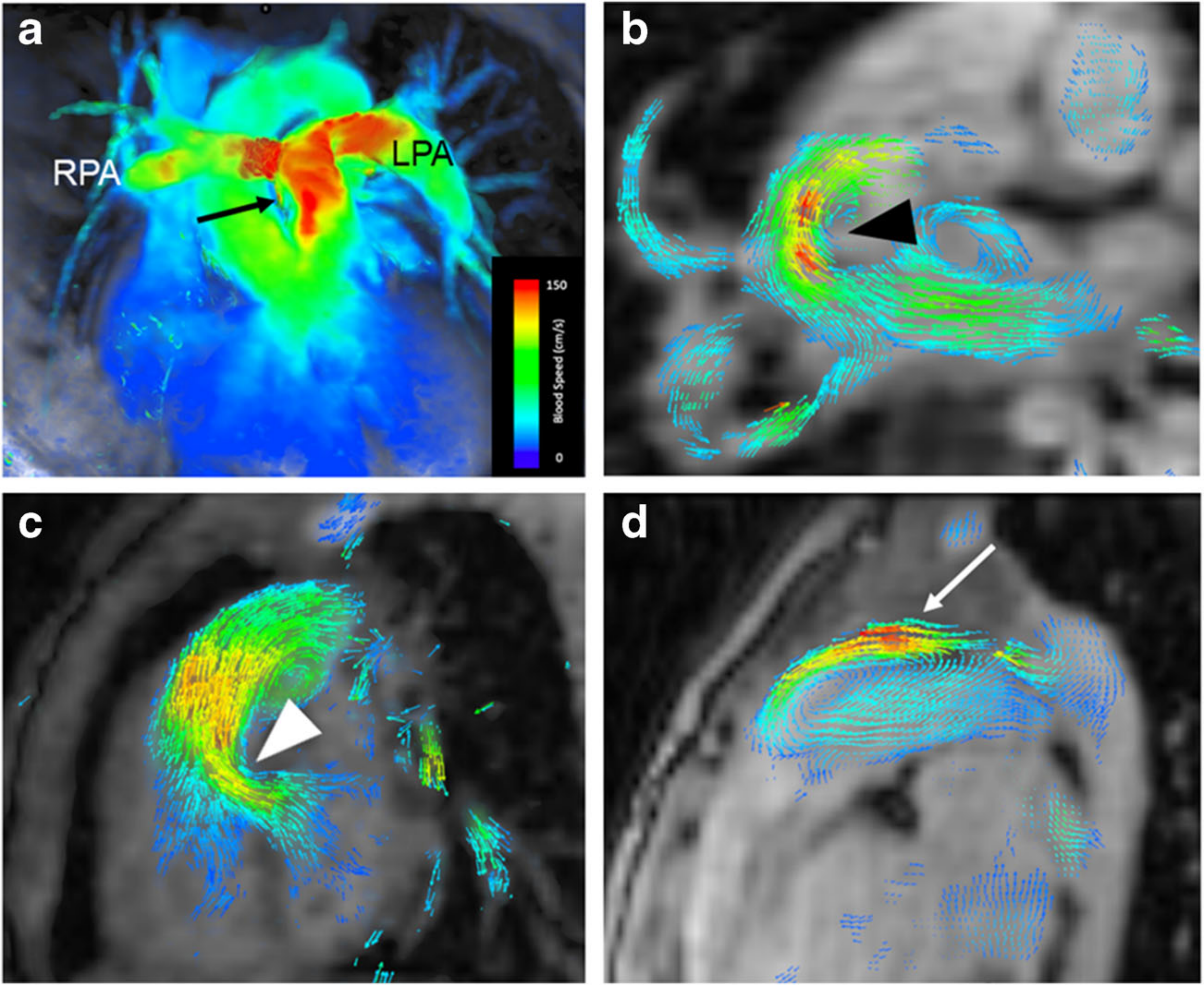
Examples of 4-D flow visualisation in four children. **a** Coronal 4-D flow velocity map in a 4-year-old girl shows accelerated flow in the main pulmonary artery (*arrow*) because of pulmonary valve stenosis that extends into the right (*RPA*) and left (*LPA*) pulmonary arteries. **b, c** Axial (**a**) and sagittal (**b**) shunt flow (*arrowhead*) through atrial (**b**) and ventricular (**c**) septal defects are visualised by colour-coded vectors in an 18-month-old girl (**b**) and a 4-day-old boy (**c**). **d** Sagittal imaging in a 2-year-old girl with patent arterial duct resulting in abnormal reverse and accelerated flow (*arrow*) in the main pulmonary artery, as visualised by colour-coded vector fields

**Fig. 2:**
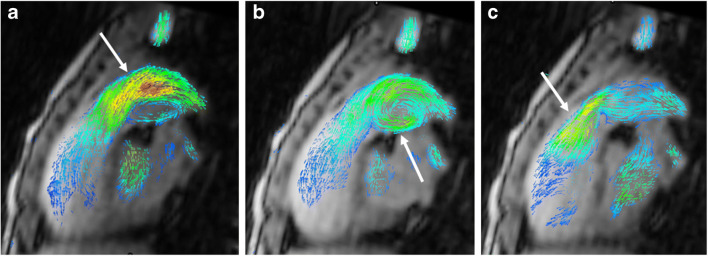
Pulmonary valve disease in a 3-year-old girl. **a–c** Sagittal 4-D flow-based dynamic vector visualisation at three different points in time during the cardiac cycle reveals antegrade systolic flow acceleration (*arrow*) caused by valve stenosis (**a**), followed by a large vortex formation (*arrow*) in the main pulmonary artery during early diastole (**b**) and retrograde flow (*arrow*) during late diastole (**c**) as a result of valve insufficiency

Flow acceleration caused by valve stenosis and retrograde flow caused by valve incompetence are easily visualised by colour-coded velocity maps, vectors or streamlines, as is shown for children with pulmonary valve stenosis (Fig. 1) and pulmonary valve insufficiency (Fig. 2). Vectors or vector fields displaying the flow direction facilitate the detection of atrial and ventricular septal defects or patent arterial ducts that are not always visible on standard cine imaging (Fig. 1). Flow direction can change during systole and diastole in septal defects and other shunts. Non-laminar, turbulent flow as a consequence of valve abnormalities or dilated vessels can be nicely appreciated by 4-D flow visualisation (Fig. 2).

**Fig. 3:**
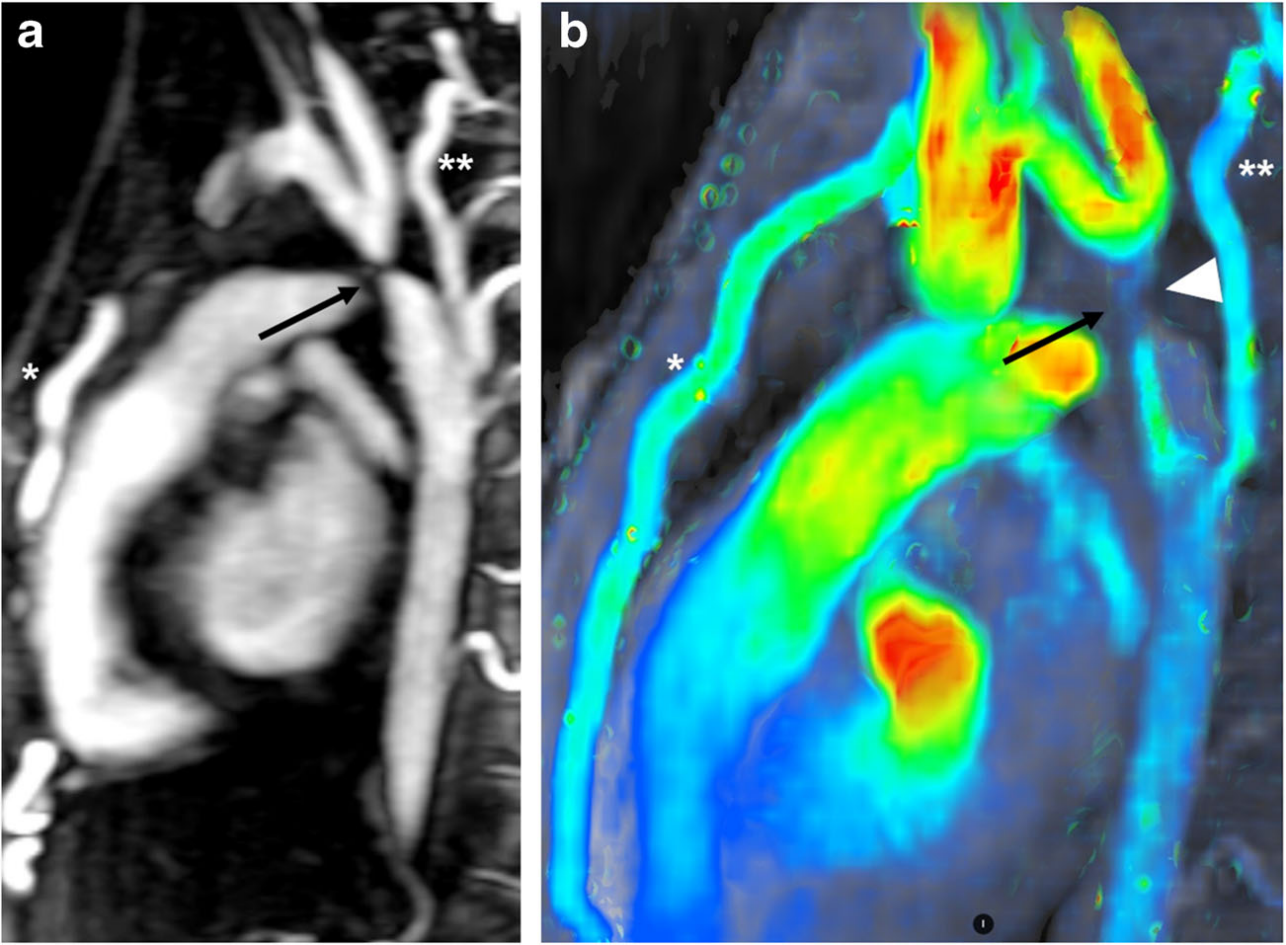
Aortic coarctation in a 4-year-old boy. Hypertension was detected at a routine medical check-up for preschool. **a** Sagittal reconstruction of magnetic resonance (MR) angiography maximum-intensity projection reveals a high-grade aortic coarctation (*arrow*) and multiple collateral arteries, mainly consisting of prominent internal mammary (*single asterisk*) and intercostal arteries (*double asterisks*). **b** Sagittal 4-D flow velocity map demonstrates lack of residual flow at the coarctation level (*arrow*). The slight blue structure is a small mediastinal collateral vessel with slow flow (*arrowhead*). The anterior (*single asterisk*) and posterior (*double asterisks*) thoracic collateral arteries are also depicted

In children with aortic pathologies, conventional sequences might fail to visualise flow in high-grade stenoses such as aortic coarctation (Fig. 3) or in vascular rings with potentially stenotic or atretic segments. Four-dimensional flow visualisation helps in determining whether there is vascular occlusion or residual flow is present. This information might have an impact on the interventional or surgical approach for the paediatric cardiologists and cardiac surgeons. In addition, collateral arteries in aortic coarctation are well visualised using 4-D flow MRI (Fig. 3).

**Fig. 4:**
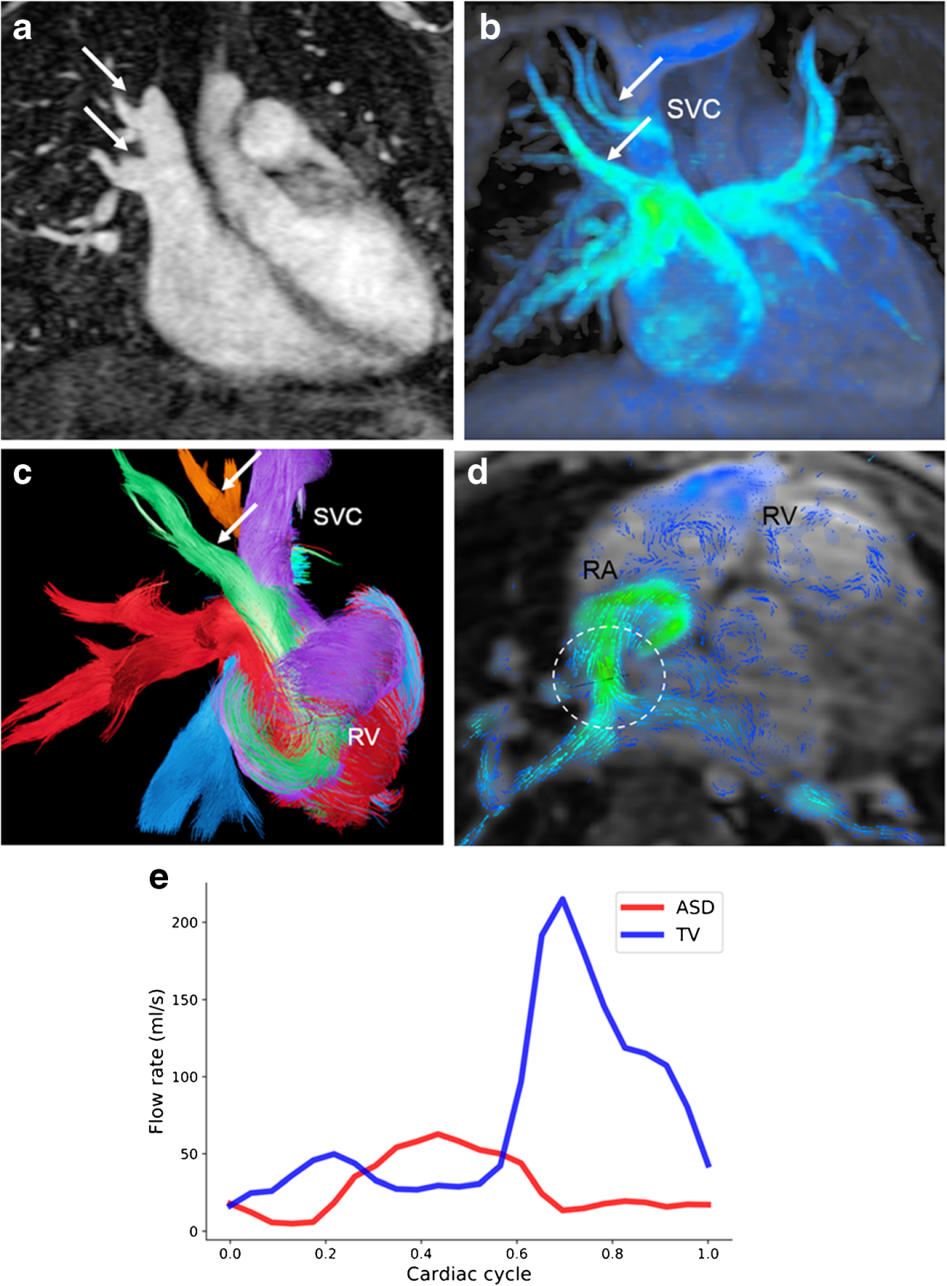
Partial anomalous pulmonary venous connection with superior sinus venosus atrial defect in a 2-year-old boy. Cardiac MRI was performed for assessment of detailed anatomy and measurement of the pulmonary-to-systemic blood flow ratio. There is a haemodynamically relevant left-to-right shunt with a calculated ratio of about 3 based on ventricular stroke volumes. **a, b** Similar to the coronal magnetic resonance (MR) angiography image (**a**), coronal 4-D flow velocity map (**b**) depicts the abnormal drainage of the right upper lobe veins (*arrows*) into the superior caval vein (*SVC*). **c** Right oblique 4-D flow-based color-coded pathline tracking shows that blood originating from the right lower and left pulmonary veins (*red*) directly passes the atrial septal defect together with blood coming from the abnormal draining right upper pulmonary veins (*arrows; orange, green*) and the SVC. **d, e** Axial 4-D flow allows for visualisation of the shunt direction by vectors (**d**) and direct shunt quantification (*dotted circle*) as shown in the diagram (**e**) for the sinus venosus defect and the tricuspid valve. *RA* right atrium, *RV* right ventricle

**Fig. 5:**
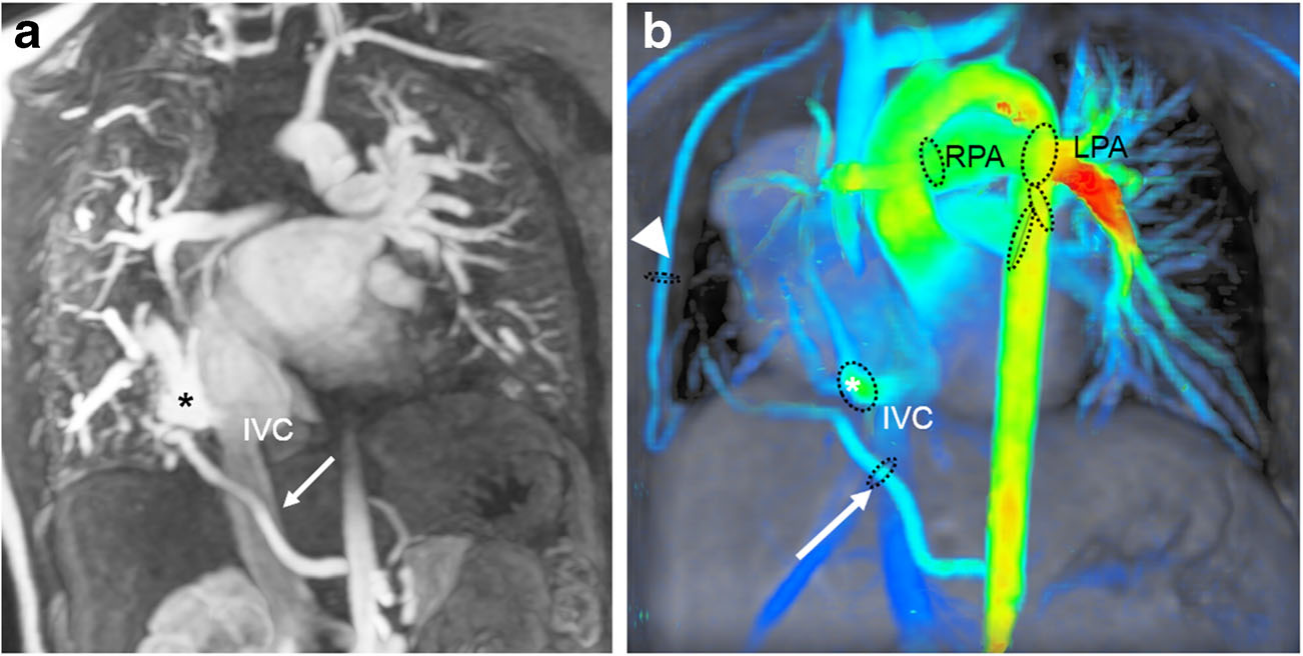
Scimitar syndrome in a 2-year-old girl. **a** Coronal reconstruction of magnetic resonance (MR) angiography maximum-intensity projection reveals the anomalous drainage of all right pulmonary veins via the so-called scimitar vein (*asterisk*) into the right atrium through the suprahepatic segment of the inferior caval vein (*IVC*). The systemic arterial supply via a feeder originating from the abdominal aorta (*arrow*) is also depicted. **b** Comprehensive flow quantification is feasible in this child by measuring the flow volume in the anomalous draining right pulmonary vein and the anomalous systemic arteries. Coronal 4-D flow velocity map gives a comprehensive overview of the vascular anomalies and shows multiple sites where flow is quantified: scimitar vein (*asterisk*), systemic feeding artery from the abdominal aorta (*arrow*), systemic feeding artery from right subclavian artery (*arrowhead*), pulmonary arteries and left pulmonary veins (*black ovals*). *LPA* left pulmonary artery, *RPA* right pulmonary artery

**Fig. 6:**
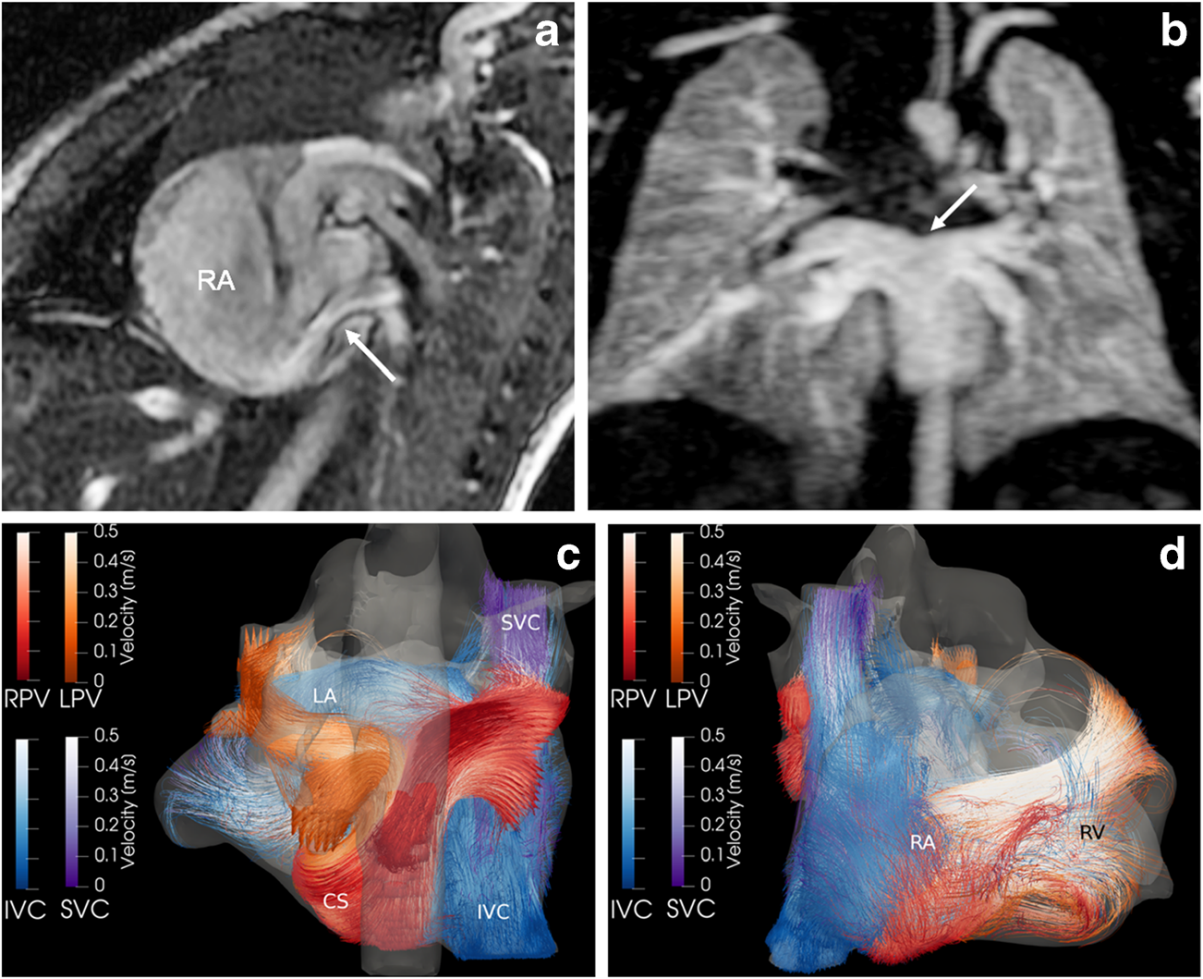
Intracardiac total anomalous venous connection in 7-day-old boy. **a** Sagittal cine steady-state free precession image (repetition time/echo time 4.2/1.8 ms) reveals the unobstructed pulmonary venous flow via a common confluence and the coronary sinus (*arrow*) into the dilated right atrium (*RA*). **b** Coronal magnetic resonance (MR) angiography maximum-intensity projection depicts the common pulmonary venous drainage (*arrow*) without obstruction. **c, d** Posterior (**c**) and anterior (**d**) views of 4-D flow-based colour-coded pathline tracking demonstrate the abnormal flow of right (RPV, *red*) and left (LPV, *orange*) pulmonary veins entering the right atrium (*RA*) inferiorly via the coronary sinus (*CS*). Blood from the inferior caval vein (*IVC*) flows directly to the left atrium (*LA*) via an associated atrial septal defect. *RV* right ventricle, *SVC* superior caval vein

**Fig. 7:**
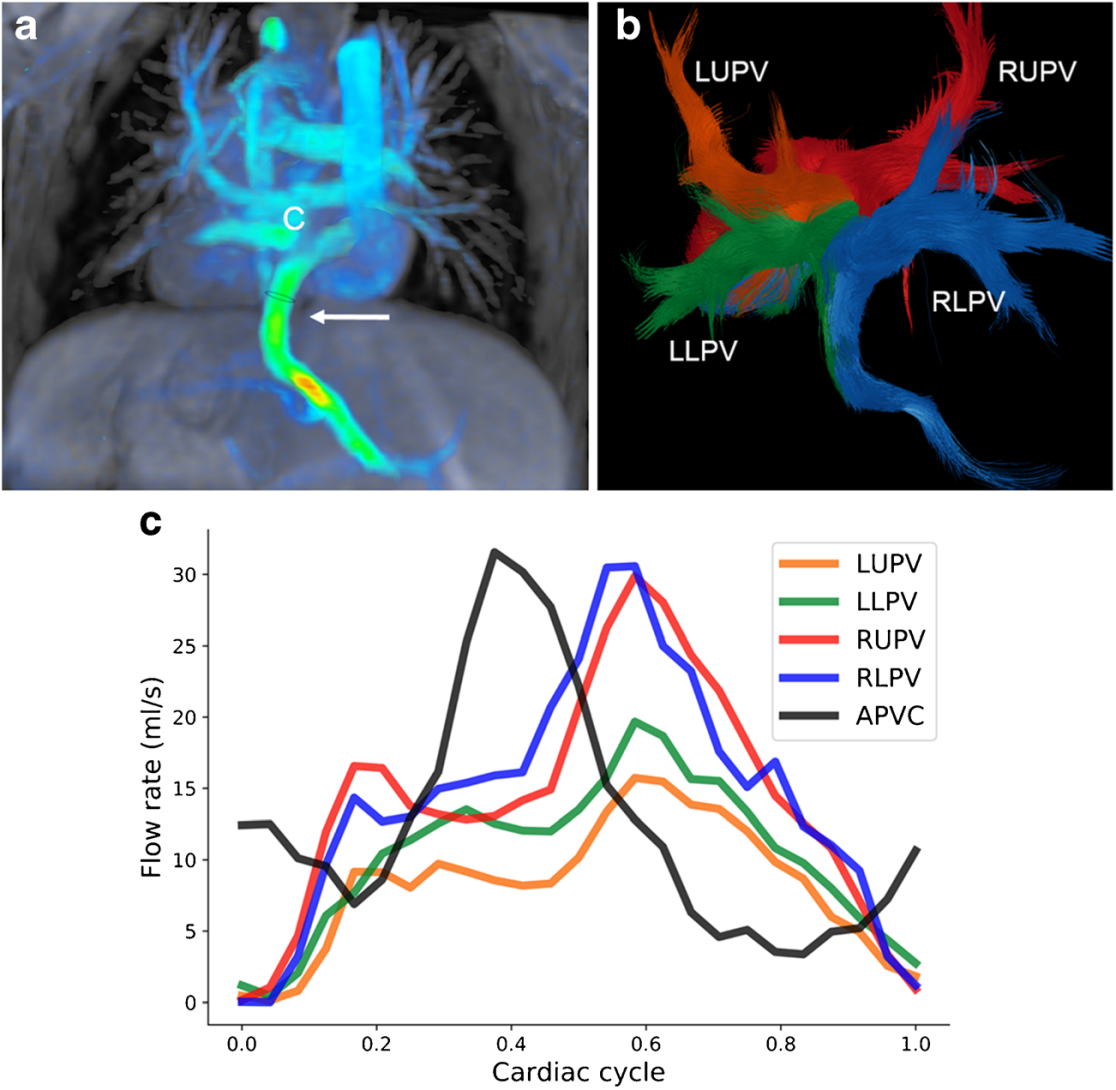
Infracardiac total anomalous pulmonary venous connection after repair with a surgical connection between retrocardiac collector and left atrium, but without ligation of the vertical vein, in a 4-year-old boy. **a** The abnormal anatomy and flow can easily be appreciated by the coronal 4-D flow velocity map depicting the collector (*C*) and the vertical vein (*arrow*) going down to the liver (posterior view). **b, c** Coronal 4-D flow-based pathline tracking with quantitative assessment reveals that about one-third of the pulmonary venous blood drains into the portal venous system, mainly from the lower lobes (*blue* and *green*). *APVC* atrial pulmonary venous connection, *LLPV* left lower pulmonary vein, *LUPV* left upper pulmonary vein, *RLPV* right lower pulmonary vein, *RUPV* right upper pulmonary vein

In young children presenting with partial or total anomalous pulmonary venous connection, colour-coded pathline tracking is extremely valuable to follow the blood direction and movement from a defined starting point. Or, more specifically, 4-D flow pathline tracking enables understanding of unusual anatomical and haemodynamic situations of the abnormal pulmonary venous drainage before and after surgery (Figs. 4, 5, 6 and 7). Additional data are given in Online Supplementary Material 1.

### Flow quantification

Quantification of an intracardiac shunt usually consists of calculating the systemic blood flow in the ascending aorta and the pulmonary flow in the main pulmonary artery, or in the branch pulmonary arteries to obtain the pulmonary versus systemic flow ratio. Another important advantage of 4-D flow compared to 2-D flow acquisitions is the possibility to sample data for measurements in different vessels under the same physiological conditions. In addition, 4-D flow MRI allows direct flow measurement of a shunt through an atrial or ventricular septal defect. In the presence of multiple shunt levels, haemodynamic understanding can be improved by adding any necessary flow quantification. This is particularly useful in young children presenting with partial anomalous pulmonary venous connection and associated superior sinus venosus atrial septal defect (Fig. 4) or in children with scimitar syndrome (Fig. 5).

In neonates and young children with total anomalous pulmonary venous connection, 4-D flow MRI gives an overview of abnormal flow paths pre- and post-surgically in different types of anomalous venous return (Figs. 6 and 7). Moreover, shunt quantification and shunt contribution of each pulmonary vein can be achieved by pathline tracking, similarly to that in children with partial anomalous pulmonary venous connection (Fig. 7).

### Individualised flow analysis in complex congenital heart disease

Four-dimensional flow assessment provides beneficial information in unique cases of complex CHD and in postsurgical conditions for assessment of residual or secondary pathologies. Among these are children with tetralogy of Fallot, transposition of the great arteries, pulmonary or tricuspid atresia and surgical stages for hypoplastic left heart syndrome (Norwood II or total cavopulmonary connection). Whereas most children after correction of tetralogy of Fallot and transposition of the great arteries are examined during adolescence or early adulthood, other cardiac defects need earlier follow-up examinations. This is particularly true for staged procedures to evaluate residual findings and monitor changes in vessel size or flow distribution.

**Fig. 8:**
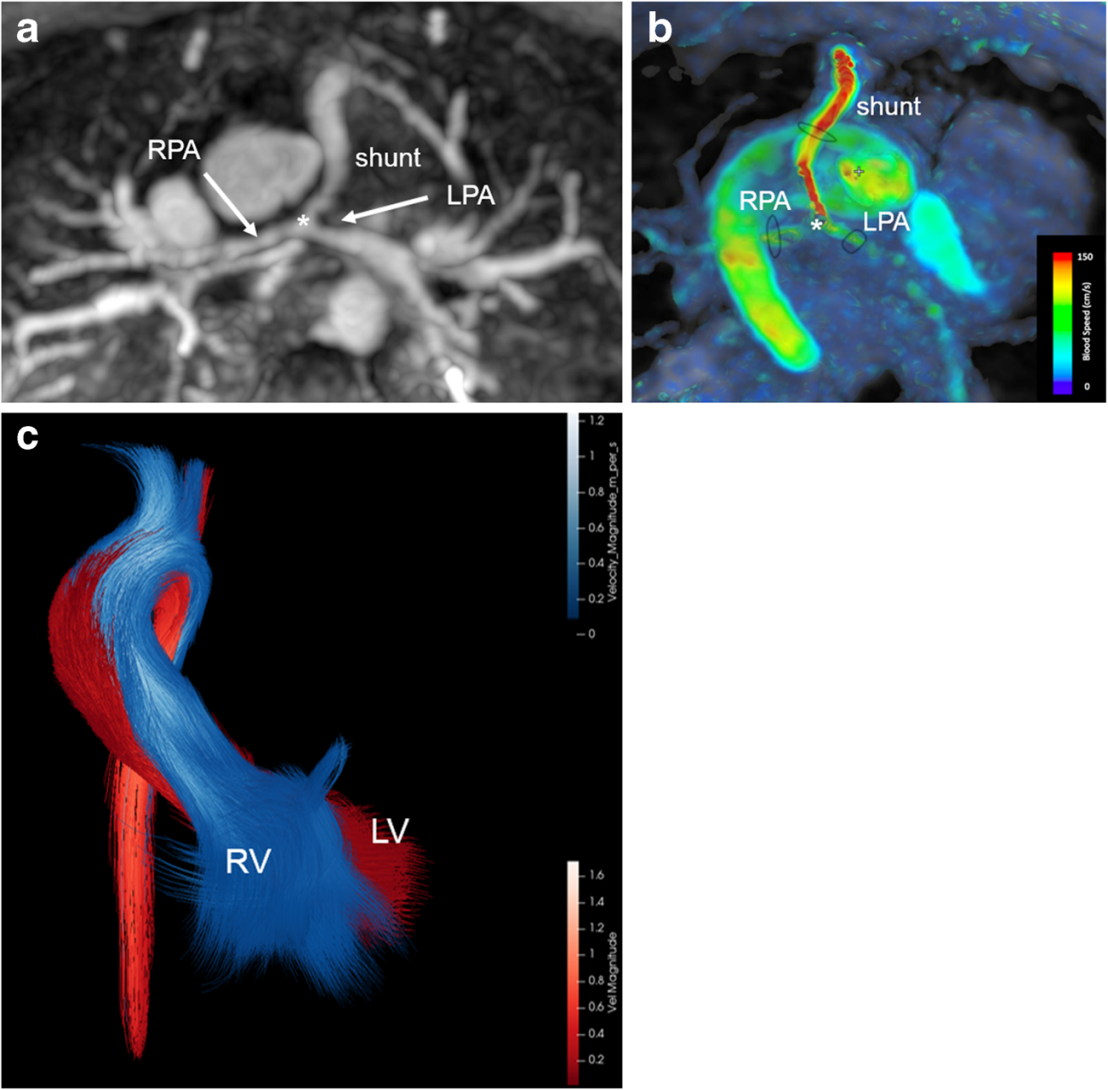
Pulmonary atresia, ventricular septal defect, confluent pulmonary arteries and multiple major aortopulmonary collaterals in an 18-month-old girl after shunt placement between right ventricle and pulmonary artery bifurcation. **a** Axial magnetic resonance (MR) angiography maximum-intensity projection shows a small-diameter shunt with distal shunt stenosis at the bifurcation (*asterisk*) as well as a tiny right pulmonary artery (*RPA*) and a proximally stenotic left pulmonary artery (*LPA*). **b** Corresponding axial 4-D flow velocity map reveals high velocities within the shunt (*red*). Flow assessment is feasible in all three arteries (*black ovals*). **c** Coronal 4-D flow colour-coded pathlines demonstrate blood flow separation of the right (*RV*) and left (*LV*) ventricles without real mixing despite the ventricular septal defect: RV blood (*blue*) mainly runs into the brachiocephalic trunk and left carotid artery, whereas LV blood (*red*) flows into the left subclavian artery

**Fig. 9:**
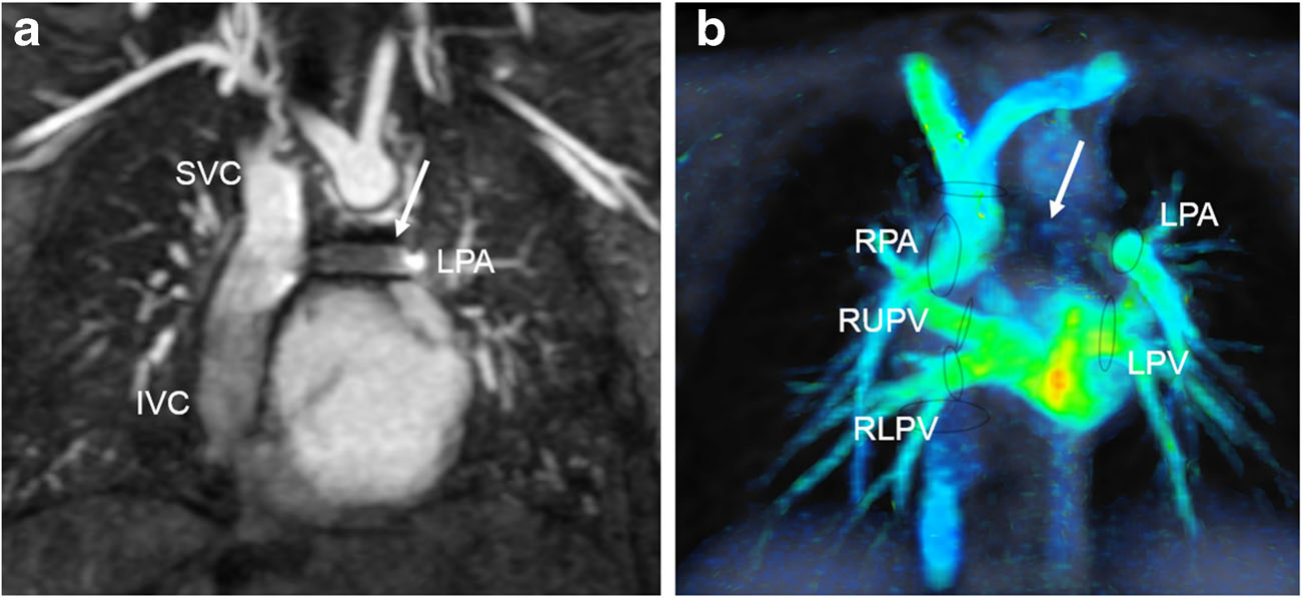
Bidirectional cavopulmonary anastomosis in a 4-year-old boy with single ventricle caused by tricuspid atresia and pulmonary stenosis. **a** Coronal magnetic resonance (MR) angiography maximum-intensity projection demonstrates patency of the stent (*arrow*) in the left pulmonary artery (*LPA*). *IVC* inferior caval vein, *SVC* superior caval vein. **b** Coronal 4-D flow velocity map gives an overview of the postsurgical anatomy. Flow within the stent (*arrow*) is not visualised. However, simultaneous flow quantification (*black ovals*) in the pulmonary arteries and pulmonary and systemic veins is more precise than 2-D phase-contrast measurements regarding flow conservation of mass because of an early branching of the right pulmonary artery at the site of the cavopulmonary anastomosis and the presence of a stent in the LPA. *LPV* left pulmonary veins, *RLPV* right lower pulmonary vein, *RPA* right pulmonary artery, *RUPV* right upper pulmonary vein

**Fig. 10:**
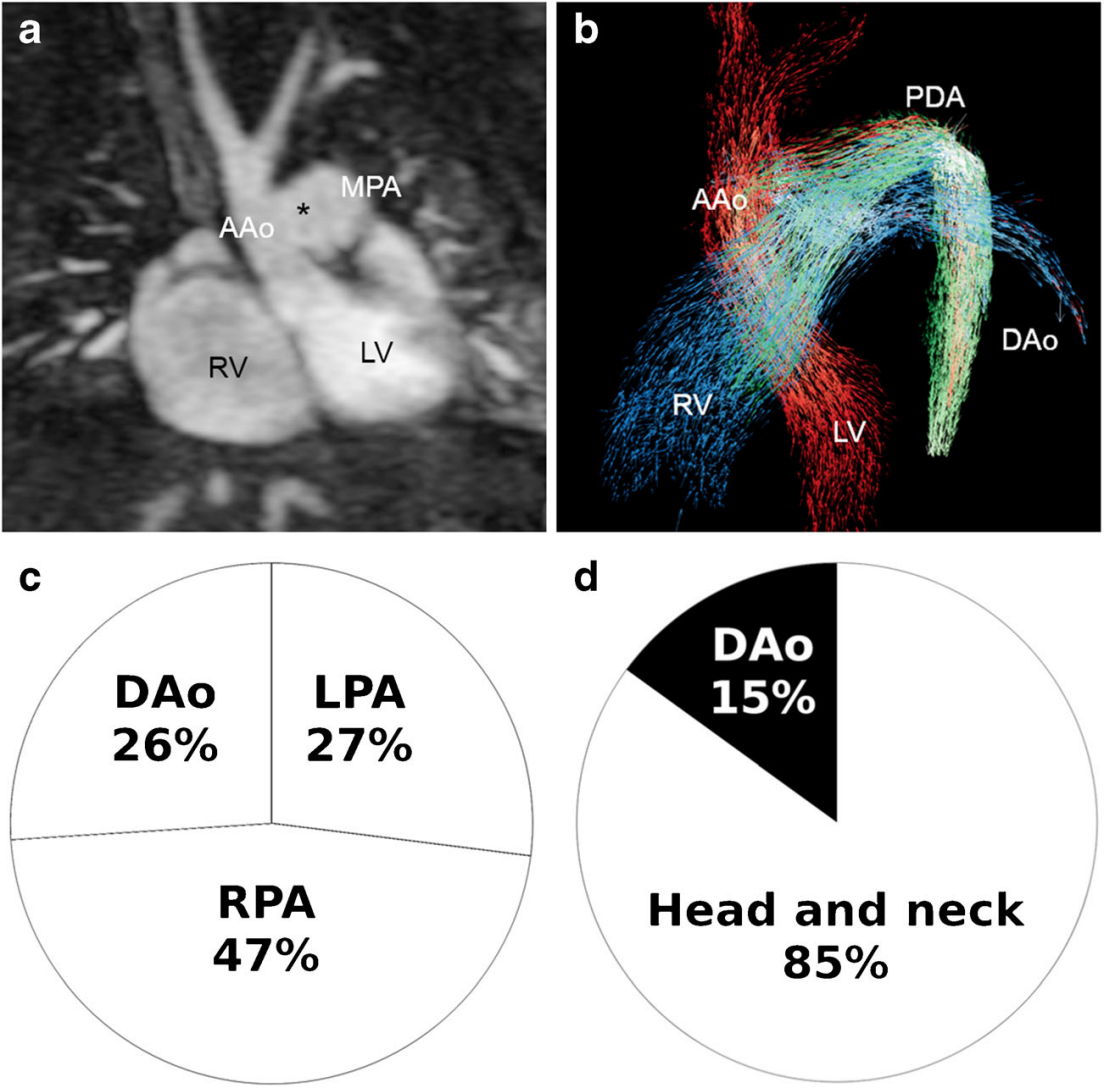
Interrupted aortic arch Type A with a large patent duct and aorto-pulmonary window in a 3-day-old girl. **a** Coronal magnetic resonance (MR) angiography image shows the large aorto-pulmonary window (*asterisk*) between the ascending aorta (*AAo*) and the main pulmonary artery (*MPA*). *LV* left ventricle, *RV* right ventricle. **b** Left oblique 4-D flow vector visualisation reveals mixing of aortic flow (*red*) and pulmonary flow (*blue*) through the large aorto-pulmonary window (*green*) and blood flow via the patent duct (*PDA*) to the descending aorta (*DAo*). **c, d** Four-dimensional flow pathline tracking analysis demonstrates that right ventricular stroke volume is divided into the pulmonary arteries and the DAo somewhat equally (**c**), whereas most of the left ventricular stroke volume goes into the head and neck vessels and only 15% into the descending aorta (DAo) via the patent duct (**d**). *LPA* left pulmonary artery*, RPA* right pulmonary artery

**Fig. 11:**
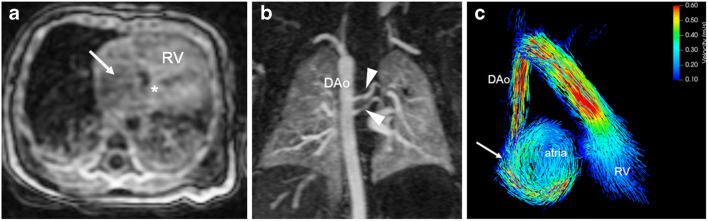
Heterotaxy syndrome associated with complete atrioventricular septal defect, insufficiency of the common atrioventricular valves, transposition of the great arteries, pulmonary atresia with non-confluent pulmonary arteries and multiple systemic collateral arteries originating from the descending aorta in a 1-day-old girl. Cardiac MRI was necessary for comprehensive anatomical overview preoperatively. **a** Axial 4-D flow MRI magnitude image demonstrates the quite impressive quality of 4-D flow raw magnitude images in neonates, resembling that of cine steady-state free precession sequences. It depicts the septal defect (*asterisk*), the right ventricular dilatation (*RV*) and a jet (*arrow*) in the right atrium caused by atrioventricular valve insufficiency. **b** Coronal MR angiography image shows two small aorto-pulmonary collateral arteries to the left lung (*arrowheads*) originating from the descending aorta (*DAo*). **c** Coronal 4-D flow pathlines reveal flow disturbances in the atria caused by insufficiency of the common atrioventricular valve resulting in a large vortex (*arrow*). The small aorto-pulmonary collaterals are not visualised

In several individual cases, which we present in Figs. 8, 9, 10 and 11 and Online Supplementary Materials 2 and 3, we demonstrate that 4-D flow is beneficial in flow volume assessment. It allows for flow volume quantification in small pulmonary arteries in which 2-D phase-contrast measurements failed (Fig. 8). It facilitates retrospective and comprehensive flow assessment, particularly in children with Fontan palliation in whom multiple evaluation planes are required (Fig. 9). In these children, differential lung perfusion can be accurately quantified by separate measurements in the pulmonary veins. Four-dimensional flow MRI enables an interactive rotation of the 3-D data and a more accurate placement of the planes for improved flow quantification, particularly in the presence of stents (Fig. 9, Online Supplementary Material 2).

Furthermore, in our experience, 4-D flow MRI allows us to analyse and understand rare cardiovascular pathologies in several unique cases in neonates with complex CHD thanks to its individualised flow visualisation and flow quantification (Figs. 10 and 11, Online Supplementary Material 3).

### Extracardiac pathologies

**Fig. 12:**
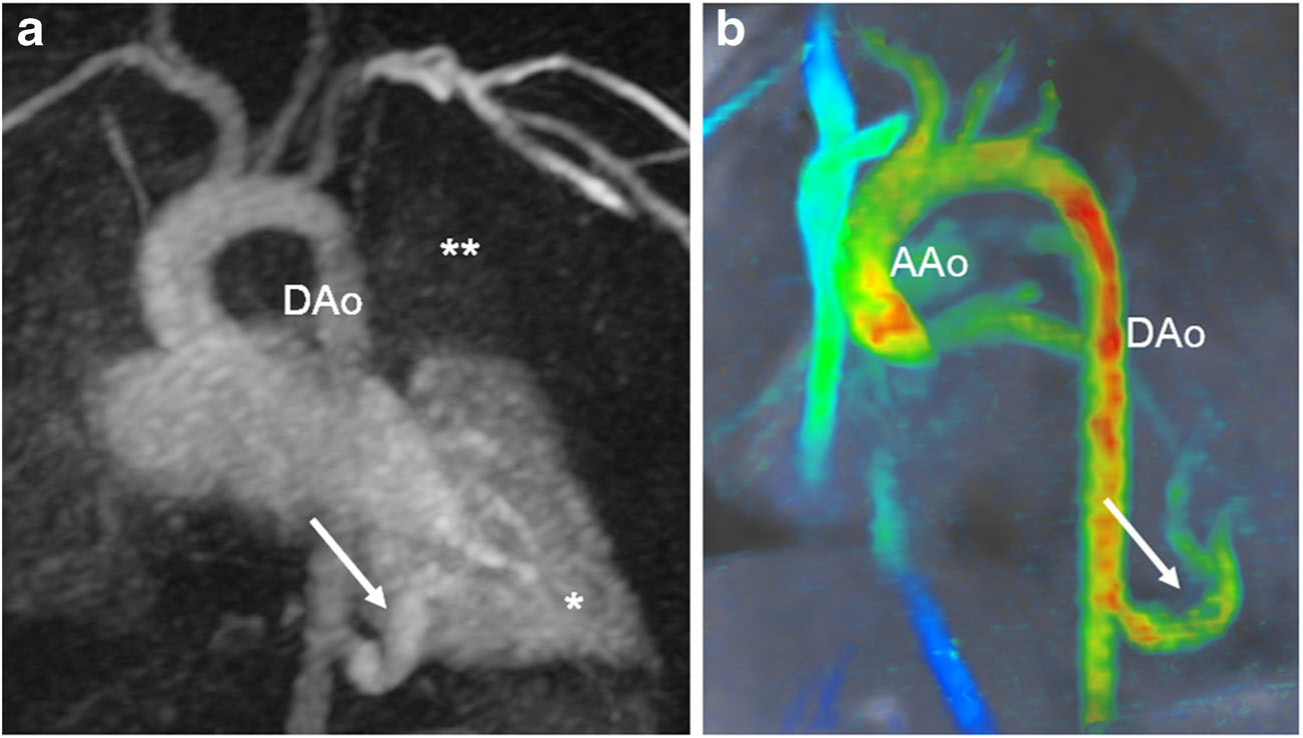
Intralobar bronchopulmonary sequestration in the left lower lobe in a 3-month-old boy. **a** Coronal image of time-resolved magnetic resonance (MR) angiography reveals the systemic artery (*arrow*) arising from the descending aorta (*DAo*) at the diaphragmatic level and the delayed pulmonary contrast enhancement (*single asterisk*) compared with the other pulmonary segments (*double asterisks*). **b** Coronal 4-D flow velocity map and flow quantification in the ascending aorta (*AAo*) and in the systemic artery (*arrow*) result in a systemic-to-pulmonary shunt of 27% of the cardiac output

There are other thoracic pathologies in which 4-D flow MRI might be contributory, including shunt quantification in bronchopulmonary sequestrations or hybrid lung lesions, as displayed in Fig. 12. Four-dimensional flow enables direct flow quantification in the systemic feeder in relation to the cardiac output.

**Fig. 13:**
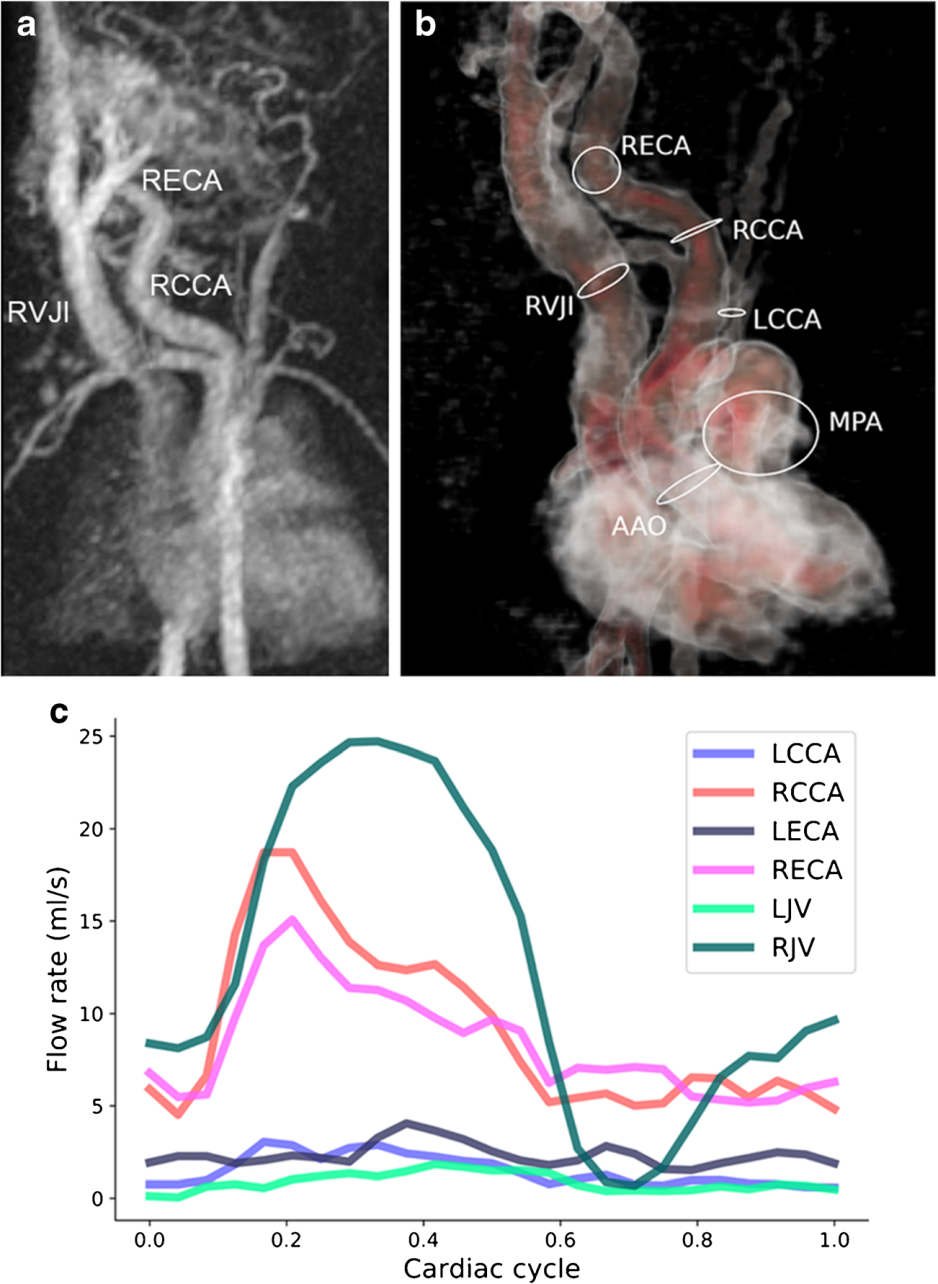
Congenital haemangioma presenting as a high-flow lesion in the neck of a 2-day-old boy. **a** Coronal image of time-resolved magnetic resonance (MR) angiography shows the dilated right common and external carotid arteries (*RCCA*, *RECA*) and enlarged internal jugular vein (*RVJI*) as well as multiple feeding and draining vessels. **b**, **c** Corresponding coronal 4-D flow vessel isosurface visualisation (**b**) and quantification (**c**) show the asymmetrical flow distribution in the right and left cervical vessels *(ovals)* because of the high-flow lesion on the right. *AAo* ascending aorta, *LCCA* left common carotid artery, *LECA* left external carotid artery, *LVJ* left internal jugular vein, *MPA* main pulmonary artery

It can also be applied for shunt quantification in vascular malformations, more specifically high-flow lesions. This is shown in a neonate with a large cervical high-flow malformation (Fig. 13), in whom vessel anatomy, flow distribution and quantification were delineated using 4-D flow before interventional treatment.

## Four-dimensional flow magnetic resonance imaging — accuracy

An internal validation of systemic versus pulmonary flow volumes in children without shunts, or of main versus combined branch pulmonary artery flow volumes, is recommended in 4-D flow datasets [40]. The conservation of mass principle also has validity for 4-D flow measurements, particularly when comparing results to 2-D phase-contrast measurements [46]. Other comparison studies point out that scanner- and setting-specific validation has to be performed for data validation at each site, in particular with regard to phantom correction [47, 48].

Several publications with focus on paediatric CHD have shown good internal validity for 4-D flow quantification in comparison with echocardiography, conventional 2-D phase-contrast measurements or cine short-axis stack ejection fractions as reference methods [22, 48–51]. Excellent correlation and agreement between 4-D flow MRI and 2-D phase-contrast results were found for net flow and regurgitant flow in the four main thoracic arteries in the study by Gabbour et al. [48]. Ventricular volumes and ejection fraction correlated well in a study by Hsiao et al. [22] comparing 4-D flow MRI with both 2-D phase-contrast and cine short-axis stack steady-state free precession results. This group could also demonstrate that the accuracy and precision of venous flow quantification are comparable to that of arterial flow quantification at velocity-encodings appropriate for arterial vessels [51]. Another study validated the internal consistency of 4-D-flow-derived volumes and demonstrated consistent measurements of net and regurgitant blood flow across the inlet and outlet valves [43].

Our experience revealed high correlation and agreement of 4-D flow data assessed by three commercially available software packages to 2-D phase-contrast data with phantom correction as a reference method [52].

## Limitations and pitfalls

The most important limitations and drawbacks of 4-D flow MRI application and assessment are still its rather long acquisition and post-processing times. Despite the use of different image acceleration methods, scan times last in the range of several minutes. Newborns are susceptible to sedation; therefore scan duration should be kept to a minimum. However, in our opinion, this prolongation of total scan time is tolerable and is compensated by the crucial additional information gained by this technique. Latest innovations in spiral and radial acquisition schemes allow for enormous acceleration and are promising developments for clinical application [33, 34].

Intracardiac devices might impair image quality comparable to other MR sequences. In general, costs, access to resources (sequence and post-processing tools) and local expertise are further limitations for a broader application of the technique.

Currently available post-processing software programmes vary in handling and visualisation or quantification options. Depending on the complexity of the case, post-processing times can last up to 1 h. However, automated processing steps, such as automated background noise correction and contour detection, help accelerate the post-processing and make it more user-friendly.

**Fig. 14:**
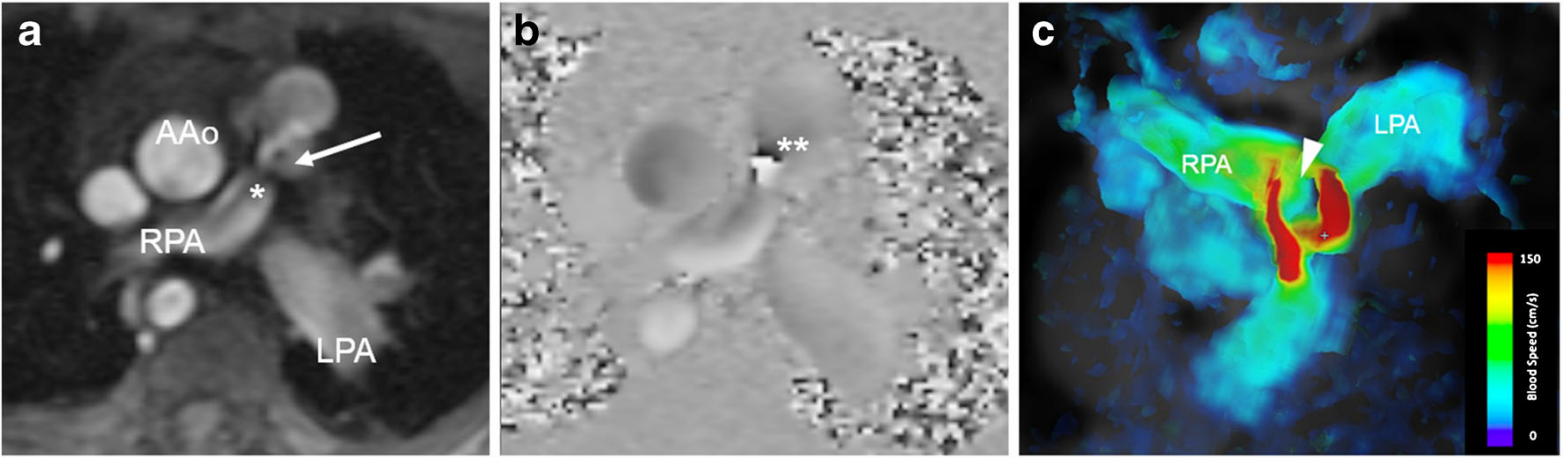
Atrioventricular septal defect after pulmonary artery banding in an 8-month-old boy. **a, b** Transverse two-dimensional phase-contrast magnitude (**a**) and phase (**b**) images (repetition time/echo time 6.7/3.7 ms) reveal stenosis at the site of pulmonary artery banding (*arrow*). This results in a localised flow jet (*single asterisk*) extending in the right pulmonary artery (*RPA*) and aliasing artifacts (*double asterisks*) at the banding site. The RPA and left pulmonary artery (*LPA*) are dilated. **c** Oblique coronal 4-D flow velocity map demonstrates the high flow velocity (*red*) at and next to the banding with post-stenotic slow flow in the dilated pulmonary arteries. There is central sparing of flow visualisation from aliasing (*arrowhead*)

In our experience a default velocity encoding of 160 cm/s is appropriate for the majority of examinations. Velocity encoding adjustments should be performed in all children with expected high-flow velocities such as valve or vascular stenoses. In cases with unclear velocities, a short 2-D phase-contrast measurement can be run before the 4-D flow acquisition to estimate the appropriate velocity-encoding setting. Otherwise, local aliasing artefacts might hamper exact flow evaluation if not corrected by the post-processing software (Fig. 14). However, velocity encoding setting should not be too high a priori, otherwise information in vessels with slow flow might get lost or be imprecise.

The current settings in acquisition and post-processing result in some limitations concerning the spatial resolution for visualising very small vessels, e.g., aortopulmonary collaterals in pulmonary atresia. Four-dimensional flow visualisation might fail to show these tiny vessels, particularly in cases with slow flow that are depicted by MR angiography (Fig. 11). Submillimetre isotropic high-resolution imaging might be achieved after clinical implementation of novel technical developments such as radial golden angle principle 3-D phase-contrast acquisitions [34].

## Outlook and future perspectives

Altered haemodynamics in children with CHD are associated with abnormalities in wall shear stress, vorticity, dissipation of intraventricular blood flow energy, changes in kinetic energy and viscous energy loss. These more advanced parameters, which can be derived from velocity-based raw data, can provide further quantitative insight into the complex interactions of cardiovascular anatomy, function and flow in children with CHD [53–57]. In the course of further research, it is likely that some of these parameters will also be available for clinical comprehensive cardiovascular MRI assessment in young children with CHD and can be applied for individual and disease-specific treatment and for predicting outcome.

In addition, recent work suggests using 4-D flow acquisitions for 3-D printing combining flow measurements, anatomical assessment and simulation for surgical planning, or using computational fluid dynamics for optimised 4-D flow applications with higher resolution, shorter scan times, and accurate quantification of physiological parameters [58, 59].

## Conclusion

As 4-D flow MRI facilitates flow acquisition and assessment in children with CHD in daily routine, it has the potential to become a clinical alternative to 2-D phase-contrast measurements. Four-dimensional flow MRI is easy to perform in neonates and young children by adequate adjustment of sequence parameters, and provides impressive image quality that can be well visualised with the currently available post-processing software. The most important advantage is the ability to retrospectively assess virtually any vessel of interest within the acquired 3-D data. We consider 4-D flow MRI as an important supplement to other MRI sequences and have implemented the sequence in our standard CHD protocols. Despite the additional scan duration and post-processing on the order of several minutes, we believe that 4-D flow, used in clinical routine in all children undergoing CMR before surgery and during follow-up, has the potential to improve individualised management and become indispensable for individual risk stratification.

## Electronic supplementary material

**Online Supplementary Material 1** Intracardiac total anomalous venous connection in 7-day-old boy (Fig. 6). Posterior view of colour-coded pathline tracking demonstrates the abnormal flow of right (RPV, *red*) and left (LPV, *orange*) pulmonary veins entering the right atrium inferiorly via the coronary sinus. Blood from the inferior caval vein (IVC, *blue*) flows directly to the left atrium via an associated atrial septal defect. *SVC* superior caval vein (AVI 9504 kb)

**Online Supplementary Material 2** Bidirectional cavopulmonary anastomosis in a 4-year-old boy with single ventricle from tricuspid atresia and pulmonary stenosis (Fig. 9). Four-dimensional flow-velocity map gives an overview of the postsurgical anatomy. Flow within the stent is not visualised. Simultaneous flow quantification (*black circles*) in the pulmonary arteries and pulmonary and systemic veins is more precise regarding flow conservation of mass (MP4 383 kb)

**Online Supplementary Material 3** Heterotaxy syndrome associated with complete atrioventricular septal defect, insufficiency of the common atrioventricular valves, transposition of the great arteries, pulmonary atresia with non-confluent pulmonary arteries and multiple systemic collateral arteries originating from the descending aorta in a 1-day-old girl (Fig. 11). Cardiac MRI was necessary for comprehensive anatomical overview preoperatively. Colour-coded 4-D flow pathlines reveal flow disturbances in the atria caused by insufficiency of the common atrioventricular valve resulting in a large vortex. Note that the girl has a right aortic arch (AVI 10433 kb)
